# Indolocarbazoles as host-directed therapeutics against intracellular infections by methicillin-resistant *Staphylococcus aureus*

**DOI:** 10.1093/jac/dkaf198

**Published:** 2025-07-01

**Authors:** Robin H G A van den Biggelaar, David Erdkamp, Adriëtte W de Visser, Susan J F van den Eeden, Anno Saris

**Affiliations:** Leiden University Center for Infectious Diseases, Leiden University Medical Center, Leiden, The Netherlands; Leiden University Center for Infectious Diseases, Leiden University Medical Center, Leiden, The Netherlands; Leiden University Center for Infectious Diseases, Leiden University Medical Center, Leiden, The Netherlands; Leiden University Center for Infectious Diseases, Leiden University Medical Center, Leiden, The Netherlands; Leiden University Center for Infectious Diseases, Leiden University Medical Center, Leiden, The Netherlands

## Abstract

**Background and objectives:**

Methicillin-resistant *Staphylococcus aureus* (MRSA) is a principal contributor to mortality and morbidity attributable to antimicrobial resistance. Many MRSA strains are able to invade host cells, thereby further evading antibiotic treatment. Previously, indolocarbazole GW296115X was identified as a hit compound with host-directed activity against intracellular MRSA. This study evaluates the efficacy and selectivity of indolocarbazoles as potential host-directed therapy against intracellular MRSA. Furthermore, we assessed the interaction between indolocarbazoles and standard-of-care antibiotics.

**Methods:**

Bioluminescent *luxBADCE*-expressing MRSA was used to evaluate antimicrobial activity in planktonic cultures and HeLa cell intracellular infection models. Drug safety was assessed through lactate dehydrogenase (LDH) release and WST-1 cytotoxicity assays. The interaction between selected indolocarbazoles in combination with vancomycin or daptomycin was determined based on the Bliss independence model.

**Results:**

Indolocarbazoles GW296115X, staurosporine aglycone, CDK4 inhibitor and SB-218078 showed activity against intracellular MRSA with SB-218078 demonstrating the best potency. None of the effective compounds impaired host cell viability, although host cell metabolic activity was affected to various extents. Combining SB-218078 or GW296115X with vancomycin or daptomycin synergistically eradicated both intracellular and extracellular MRSA.

**Conclusions:**

Indolocarbazoles, particularly GW296115X and SB-218078, enhance standard antibiotic efficacy against invasive MRSA and offer potential for host-directed therapy.

## Introduction

*Staphylococcus* (*S.*) *aureus* is an opportunistic pathogen responsible for a range of diseases, from mild skin infections to severe conditions like sepsis and pneumonia. In particular, the increasing prevalence of infections by methicillin-resistant *S. aureus* (MRSA) significantly contributes to mortality and morbidity, thereby posing a major threat to healthcare systems.^1,2^ Many MRSA strains are highly invasive, capable of penetrating host cells to evade both the immune system and antibiotic treatment.^3–8^ Because MRSA is resistant to beta-lactam antibiotics, which are the standard treatment for *S. aureus* infections, it is typically treated with vancomycin, although several alternatives like linezolid or daptomycin may be used as well. Notably, vancomycin performs poorly against intracellular MRSA, despite its accumulation intracellularly, for reasons not yet fully understood.^3,5,9^ This allows MRSA to persist within cells, potentially leading to recurrent infections once antibiotic therapy is discontinued, and contributes to the emergence of new resistance patterns like vancomycin-resistant *S. aureus* (VRSA).^10^ Adjunctive host-directed therapy (HDT) can specifically target persistent intracellular bacteria by enhancing host defense mechanisms, presenting a promising approach to improving treatment outcomes.

In a recent screen of kinase inhibitors, we identified the indolocarbazole GW296115X as an effective and highly selective HDT against MRSA, both *in vitro* and in zebrafish embryos.^11^ In parallel, others have identified GW296115X as HDT against intracellular *Mycobacterium abscessus*, suggesting broad-spectrum antimicrobial activity.^12^ GW296115X targets several kinases including PDGF, the 90 kDa ribosomal S6 kinase (RSK) family and adenosine monophosphate-activated protein kinase (AMPK)-related kinases.^11,13,14^ Interestingly, we and others have demonstrated that treatment with GW296115X paradoxically results in hyperphosphorylation of AMPK at its activation loop, which eventually seemed to stimulate MSRA degradation through autophagy.^11^ Since GW296115X was the only indolocarbazole present in the kinase inhibitor library, we wanted to investigate whether commercially available structural analogues may also serve as HDTs and perhaps be even more potent or selective. Additionally, we aimed to investigate whether indolocarbazoles act additively or synergistically with traditional antibiotics.

## Materials and methods

### Reagents

Antibiotics tested in this study included gentamicin sulphate, linezolid, tetracycline HCl and vancomycin HCl from Merck (Darmstadt, Germany) and daptomycin from Cayman Chemical Company (Ann Arbor, MI, USA). Linezolid was dissolved at 3.4 mg/mL in DMSO. Gentamicin, daptomycin and tetracycline were dissolved at 50 mg/mL in ultrapure water. Vancomycin HCl was dissolved at 10 mg/mL in ultrapure water. Compounds tested included GW296115X from AOBIOUS (Gloucester, MA, USA), staurosporine, 7-hydroxystaurosporine (UCN-01) and bisindolylmaleimide IV from Merck, staurosporine aglycone (K252c), K252a, Cdk4 inhibitor, SB-218078, Gö 6976 and arcyriaflavin A from Cayman Chemical Company.

### Cell culture

HeLa and MCF-7 cell lines were maintained in Iscove’s Modified Dulbecco’s Medium (IMDM) and Dutch-modified Roswell Park Memorial Institute (RPMI) 1640 cell culture media, respectively, supplemented with 10% foetal calf serum (FCS; Greiner Bio-One, Alphen a/d Rijn, The Netherlands) and 100 units/mL penicillin and 100 mg/L streptomycin (Gibco, ThermoFisher Scientific, Waltham, MA, USA). The cells were kept at 37°C/5% CO_2_ and passaged twice a week.

### Bacterial culture

The following *S. aureus* strains were used in this study: MRSA USA300 LAC JE2 wild type as well as transformed with pRP1195-luxBADCE,^15^ clinical isolates LUH15392 and LUH15393,^11^ and the non-invasive methicillin susceptible Newman strain.^16^ For experiments, the bacteria were recovered from a frozen glycerol stock, resuspended in Difco Mueller Hinton (MH) or tryptic soy (TS) broth (BD Biosciences, Franklin Lakes, NJ, USA) and cultured overnight at 37°C. MRSA was passaged 1:33 two to three hours prior to experiments to obtain a log-phase bacterial culture. The frozen glycerol stock of MRSA-lux was comprised of bacteria cultured in MH broth supplemented with 10 mg/L chloramphenicol (Sigma Aldrich) to select for bacteria harbouring the plasmid.

### Bacterial growth assay

The number of colony-forming units (cfu) present in the bacterial suspension was estimated from the optical density at 600 nm (OD600) by assuming 4 × 10^8^ cfu to be present in a bacterial suspension with OD600 = 1. MRSA cultures were resuspended in MH broth and added to white flat-bottom 96-well plates (Greiner) with approximately 5 × 10^4^ cfu/well together with treatments. Real-time bioluminescent measurements to observe MRSA-lux growth were performed overnight for 16 h at 37°C, using a SpectraMax i3x plate reader with an integration time set at 8000 ms.

### Intracellular infection assay

HeLa cells were resuspended in IMDM cell culture medium with 10% FCS without antibiotics, seeded with 10 000 cells/well into white/clear-bottom 96-well plates (Greiner), and were incubated overnight at 37°C/5% CO_2_. The next day, bacteria were centrifuged at 3000 *g* for 10 min and resuspended in cold PBS + 5 mM ethylenediaminetetraacetic acid (EDTA). The bacterial suspension was diluted in IMDM and approximately 2 × 10^5^ cfu/well were added to an estimated 2 × 10^4^ HeLa cells/well after overnight incubation. HeLa cells were centrifuged for 3 min at 150 *g* and incubated for 1 h at 37°C/5% CO_2_. The accuracy of each MRSA inoculum was determined by plating 10 μL spots of 10-fold dilution series on Difco tryptic soy agar plates (BD Biosciences). The plates were incubated o/n at 37°C, and cfu were counted the next day. The calculated multiplicity of infection throughout all experiments was 12.9 ± 4.0 (mean ± SD). After infection, the cells were incubated with fresh IMDM supplemented with 30 mg/L gentamicin sulphate (Lonza BioWhittaker, Basel, Switzerland) for 10–15 min at 37°C/5% CO_2_ to kill residual extracellular bacteria. Next, the infected cells were treated with IMDM containing serial dilutions of antibiotics, serial dilutions of GW296115X analogues together with 5 mg/L gentamicin (to prevent extracellular outgrowth of bacteria), or chequerboard titrations of either GW296115X or SB-218078 combined with either vancomycin or daptomycin. Prior to treatment with chequerboard titrations, the HeLa cells were washed twice with IMDM to remove any traces of gentamicin. Real-time bioluminescent measurements to observe MRSA-lux growth were performed for 16 h at 37°C, using a SpectraMax i3x plate reader with an integration time set at 8000 ms.

### LDH-release viability assay

The supernatant was collected from MRSA-infected HeLa cells after overnight treatment. Host cell viability was determined by lactate dehydrogenase (LDH)-release assay (Roche, Merck) following the manufacturer’s guidelines. As a positive control, cells were treated with triton X-100 (Merck) for 5 min to lyse the cells and induce maximum LDH release. Uninfected cells were used as the negative control. The absorbance was measured using the SpectraMax i3x at 485 and 690 nm, the latter as reference for background subtraction. The percent viable cells were calculated by: $\text{\%}\;\mathrm{viability}\;=\;100\text{\%}-\;\frac{X-cntr_{neg}}{cntr_{pos}-cntr_{neg}}*100\text{\%}.$

### WST-1 metabolic activity assay

HeLa cells were resuspended in IMDM cell culture medium with 10% FCS without antibiotics, seeded with 10 000 cells/well into transparent flat-bottom 96-well plates (Greiner), and were incubated overnight at 37°C/5% CO_2_. The next day, HeLa cells were treated with IMDM containing serial dilutions of GW296115X analogues and incubated overnight at 37°C/5% CO_2_. Untreated cells were used as control. The next day, the cell culture medium was replaced with water-soluble tetrazolium salt 1 (WST-1; Roche, Merck) reagent diluted 1:10 in IMDM. The cells were incubated for 2 h at 37°C/5% CO_2_. The cells were shaken for 1 min on a microplate shaker and the absorbance was measured at 438 and 690 nm, the latter as reference for background subtraction, using the SpectraMax i3x. Subsequently, the percentage metabolic activity was calculated by: % metabolic activity = $\frac{X-cntr_{medium}}{cntr_{untr}-cntr_{medium}}*100\text{\%}.$

### Determination of colony-forming units

HeLa and MCF7 cells infected with *S. aureus* were treated for 24 h with combinations of vancomycin, daptomycin, GW296115X and SB-218078 and subsequently lysed in sterile water + 0.05% UltraPure SDS solution (Invitrogen, ThermoFisher Scientific) to determine the remaining colony-forming units intracellularly. The lysates were 10-fold serially diluted in PBS, and 10 μL drops were plated on square TS agar plates. The plates were left to dry and incubated overnight at 37°C/5% CO_2_.

### Non-linear regression analyses

Data analysis was performed using GraphPad Prism v.10 (GraphPad Software, La Jolla, CA, USA). Dose-titration experiments were analysed by non-linear regression analyses. Relative light units (RLU) obtained by bioluminescent measurements were normalized against the negative control (untreated or DMSO-treated). The data were subjected to an extra sum-of-squares F test to examine whether four-parameter logistic regression provided a better fit than baseline (i.e. no effect). If not, the baseline was plotted instead. Half maximal effective drug concentrations (EC_50_) were determined by four-parameter logistic regression using the formula: $RLU=RLU_{min}+\;\frac{RLU_{max}-\;RLU_{min}}{1+{\left(\frac{EC_{50}}{X}\right)}^{h}}$ with *h* being the Hill coefficient. The maximum drug effect (E_max_) was calculated by: $E_{max}=\;RLU_{max}-\;RLU_{min}$. Half maximal inhibitory concentrations (IC_50_) were determined by interpolating the concentration at which the metabolic activity or host cell viability was reduced by 50% compared to the DMSO-treated controls. Outlier detection was applied to all non-linear regression analyses using the ROUT method with a maximum false discovery rate set to 1%.^17^ Approximate confidence intervals (CI) were estimated for calculated parameters.

### Evaluation antibiotic-HDT interactions

Interactions between antibiotics and HDTs were determined from bioluminescence data collected from chequerboard titrations. The growth in bioluminescent signal of MRSA-lux-infected HeLa cells (dRLU/dt) was followed over time until a maximum bioluminescent signal was reached for the untreated control. The growth in bioluminescent signal over time was averaged and normalized to the untreated controls. The SynergyFinder+ web application (www.synergyfinderplus.org) was used to determine the overall level of synergy and dose-specific levels of synergy between compounds based on the Bliss model.^18^ Heatmaps with Bliss synergy scores were created using GraphPad Prism.

### Statistical testing

Using GraphPad Prism, data were tested for significant differences between groups using a one-way ANOVA test for matched samples and Tukey’s multiple comparisons *post hoc* test. The *P* values of the overall bliss synergy scores show the statistical significance of the difference between the estimated average synergy score and the expected synergy score of zero (i.e. no interaction).^18^ Any *P* value of <0.05 was considered statistically significant.

## Results

### Several commonly used antibiotics against MRSA show limited activity against intracellular bacteria

**Figure 1.**
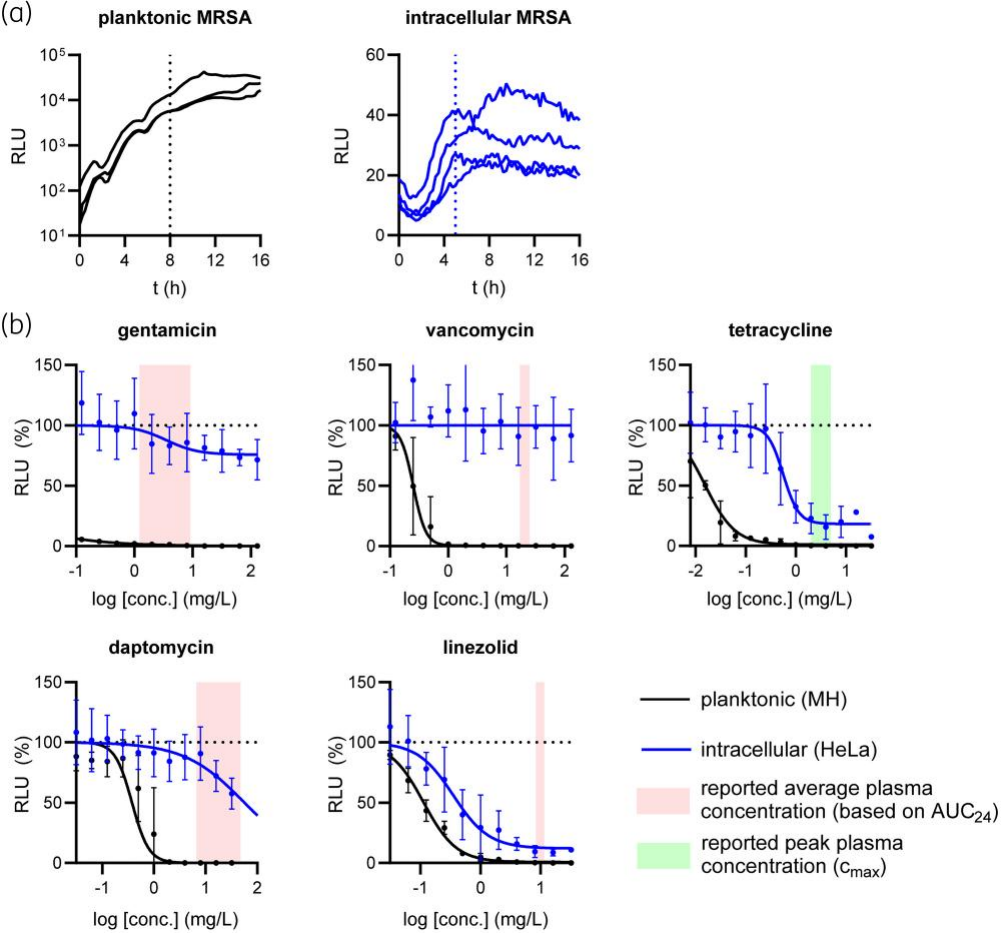
Activity of selected antibiotics against planktonic MRSA and MRSA in infected HeLa cells. (a) Real-time bioluminescent measurements of untreated planktonic (left; *n* = 3) and intracellular MRSA-lux cultures (right; *n* = 4). (b) Bioluminescent signal of planktonic (black) and intracellular (blue) MRSA-lux measured at 8 h and 5 h (see vertical dashed lines in (a)), respectively, after treatment with antibiotics at indicated concentrations. RLU data were normalized to untreated controls (horizontal dashed lines). Circles and error bars represent the mean ± SD. The curves show the result of four-parameter logistic regression analyses. For reference, pink and green areas show average and peak plasma concentrations in treated patients, respectively, as reported in the EUCAST Rationale Documents.^19^

First, we evaluated the effectiveness of commonly used antibiotics against extracellular planktonic bacteria as well as intracellular bacteria in HeLa cells using a bioluminescent MRSA reporter strain. Bioluminescence allows real-time, *in situ* quantification of the (intracellular) bacterial burden and shows a strong correlation with cfu counts during (intracellular) exponential growth (Figure S1, available as Supplementary data at *JAC* Online). The antibiotics selected for this study included gentamicin, vancomycin, tetracycline, daptomycin and linezolid. The activity of antibiotics against planktonic MRSA was assessed 8 h after treatment, just before the bacterial growth curves reached the plateau phase (Figure 1a). For intracellular MRSA, antibiotic activity was evaluated 5 h post-infection, a timepoint at which bacteria were still present intracellularly as confirmed by microscopic examination. As expected, all antibiotics demonstrated high activity and potency against planktonic MRSA, resulting in complete elimination of bioluminescent signal at relatively low concentrations (Figure 1b). Importantly, the reduction in bioluminescent signal is not due to decreased translation of lux luciferase—as might be expected with translation-inhibiting antibiotics like tetracycline and linezolid—but instead closely mirrors a decline in bacterial growth (Figure S2). In contrast to their activity against planktonic MRSA, significant differences were observed between antibiotics in their ability to target intracellular MRSA. Gentamicin and vancomycin showed limited (E_max_ = 24.2% reduction [95% CI: 11.0%–37.5%]) and no activity against intracellular bacteria, respectively. Furthermore, while tetracycline exhibited strong activity against intracellular MRSA (E_max_ = 81.7% [95% CI: 69.6%–93.9%]), the potency against intracellular bacteria was markedly reduced in comparison to its potency against planktonic cultures (EC_50_ = 562 μg/L [95% CI: 377–747 μg/L] versus EC_50_ = 15.4 ng/mL [95% CI: 14.1–16.7 μg/L]). The same was observed for daptomycin, for which its potency against intracellular cultures was at least 100 times weaker than for planktonic cultures (EC_50_ = 377 μg/L [95% CI: 322–431 μg/L]). In contrast, linezolid maintained a high level of activity against intracellular bacteria (E_max_ = 87.9% reduction [95% CI: 78.5%–99.4%]), and its potency against intracellular and extracellular bacteria was comparable (EC_50_ = 354 μg/L [95% CI: 212–495 μg/L] versus EC_50_ = 113 μg/L [95% CI: 103–123 μg/L]), highlighting its potential as a more effective option to target intracellular MRSA. These findings demonstrate that many antibiotics commonly used against MRSA show no or limited activity against intracellular bacteria, underscoring the potential benefit for HDT as part of combination therapies.

### Several indolocarbazoles are effective as HDTs against intracellular bacteria with SB-218078 being more potent than GW296115X

**Figure 2.**
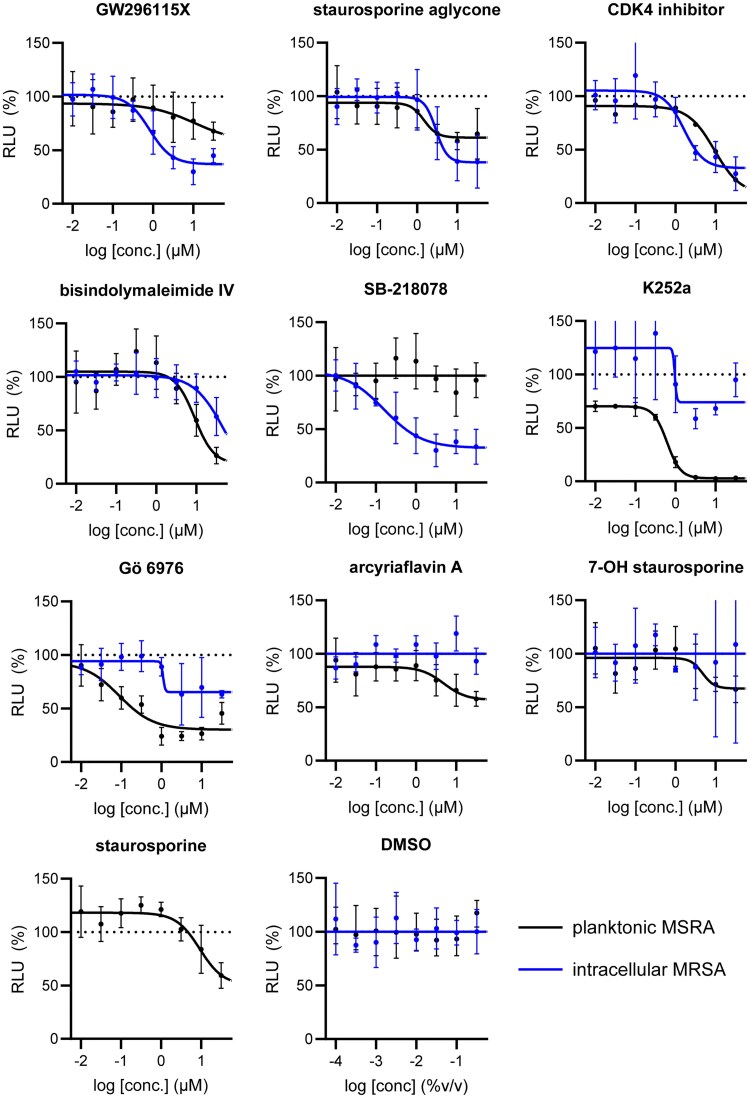
Activity of GW296115X analogues against planktonic MRSA and MRSA in infected HeLa cells. Bioluminescent signal of planktonic (black) and intracellular (blue) MRSA-lux measured 8 and 5 h, respectively, after treatment with indolocarbazoles at indicated concentrations. RLU data were normalized to DMSO controls. Datapoints and error bars represent the mean ± SD. The curves show the result of four-parameter logistic regression analyses. For staurosporine, the bioluminescent data for intracellular MRSA were out of range (>200%) and is therefore not depicted.

Building on our previous identification of GW296115X as an effective HDT against intracellular MRSA,^11^ we set out to investigate whether structural analogues exhibit similar activity against intracellular MRSA. To this end, we tested eight indolocarbazoles, in addition to GW296115X, including staurosporine aglycone, CDK4 inhibitor, SB-218078, K252a, Gö 6976, arcyriaflavin A, 7-OH staurosporine and staurosporine, as well as bisindolylmaleimide IV. Of these, four compounds—including staurosporine aglycone, CDK4 inhibitor, bisindolylmaleimide IV and SB-218078—demonstrated similar activity against intracellular *S. aureus* as GW296115X, reducing the intracellular bacterial burden with approximately 60%–70%, albeit with varying potencies (Figure 2; Table 1). GW296115X (EC₅₀ = 0.813 µM) was more potent than CDK4 inhibitor (1.63 µM), staurosporine aglycone (2.94 µM), or bisindolylmaleimide (>31.6 µM). However, SB-218078 displayed significantly higher potency than GW296115X, indicating a strong inhibitory effect on intracellular MRSA survival. Moreover, K252a and Gö 6976 showed some activity against intracellular MRSA, but the results were less clear and not as consistent as the other active compounds. Arcyriaflavin A and 7-OH staurosporine showed no activity against intracellular bacteria, while staurosporine was even host detrimental, leading to a 1.6–4.3-fold increase in bioluminescence at the concentrations tested (data not shown).

**Table 1.** Summary of estimated activity and cytotoxicity parameters of GW296115X analogues

Compound	Structure	Activity against intracellular MRSA (t = 5 h)		Activity against planktonic MRSA (t = 8 h)		WST-1 (w/o MRSA)	LDH assay (with MRSA)
		E_max_ (%)	EC_50_ (μM)	E_max_ (%)	EC_50_ (μM)	IC_50_ (μM)	IC_50_ (μM)
GW296115X	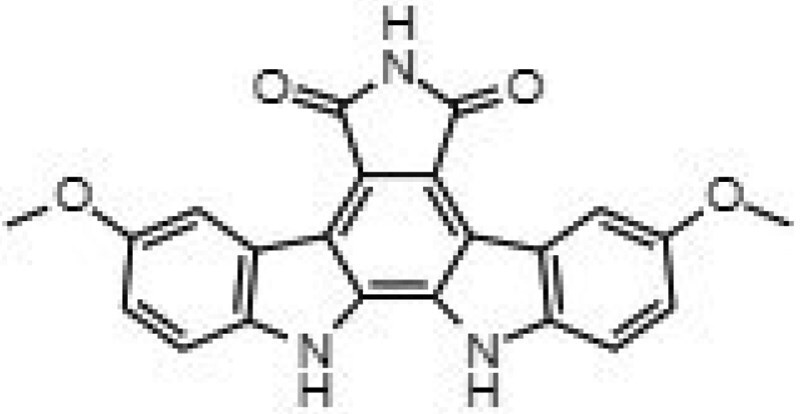	64.8 [48.4, 81.2]	0.813 [0.311, 1.32]	36.4 [∼0, ∼100]	10.8 [<0.01, >32]	>32	>32
staurosporine aglycone	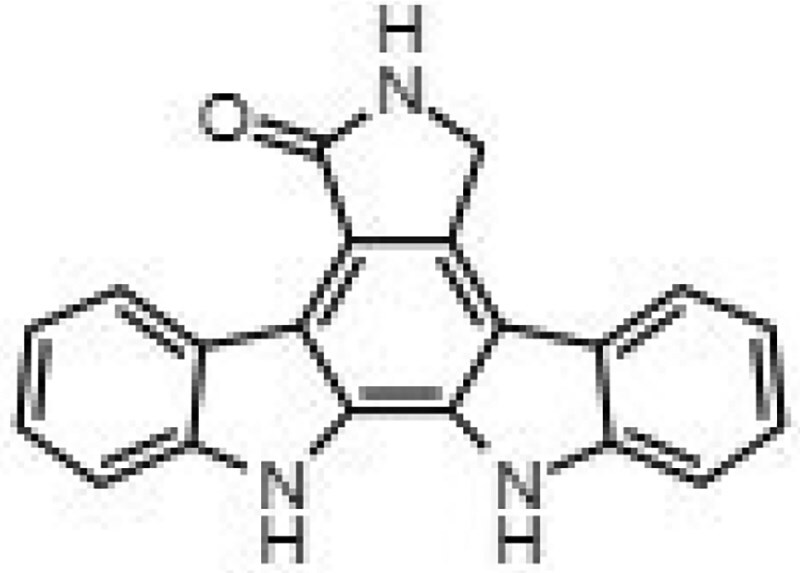	61.2 [41.6, 80.8]	2.94 [1.54, 4.33]	32.6 [14.0, 51.3]	1.48 [<0.01, 3.46]	>32	>32
CDK4 inhibitor	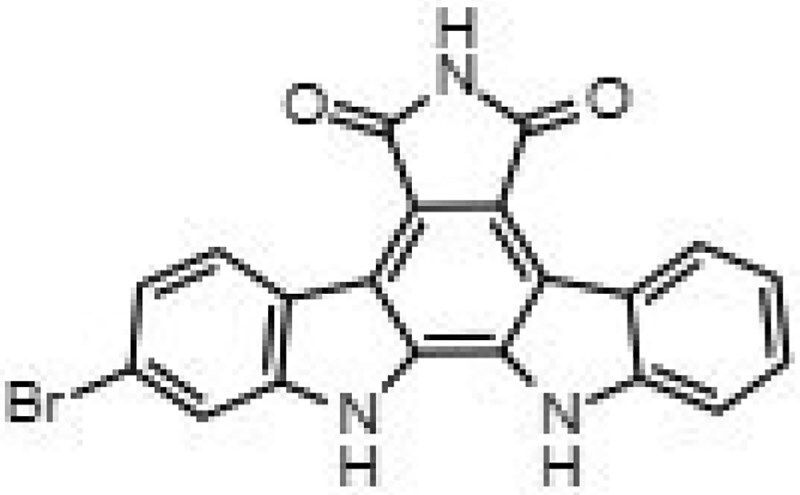	72.5 [49.2, 95.9]	1.63 [0.404, 2.85]	82.8 [22.3, ∼100]	9.42 [<0.01, 22.5]	>32	>32
bisindolylmaleimide IV	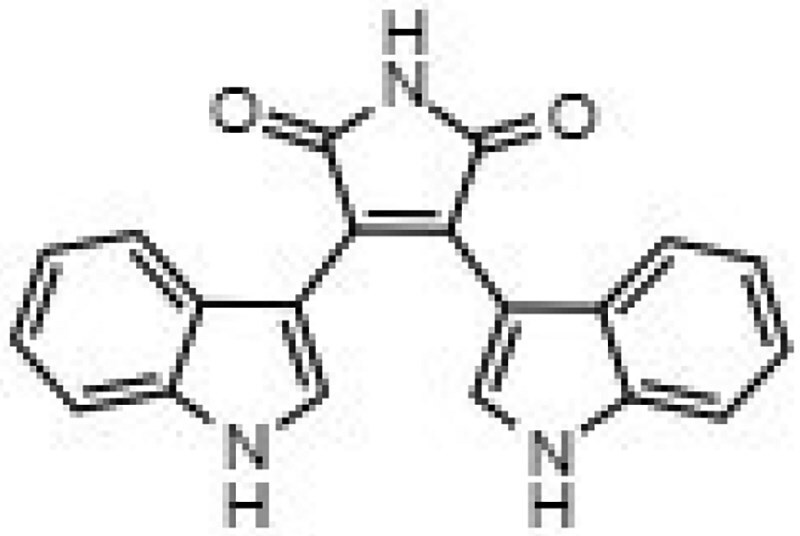	≥37.2	>31.6	86.3 [25.1, ∼100]	9.07 [<0.01, 19.2]	>32	>32
SB-218078	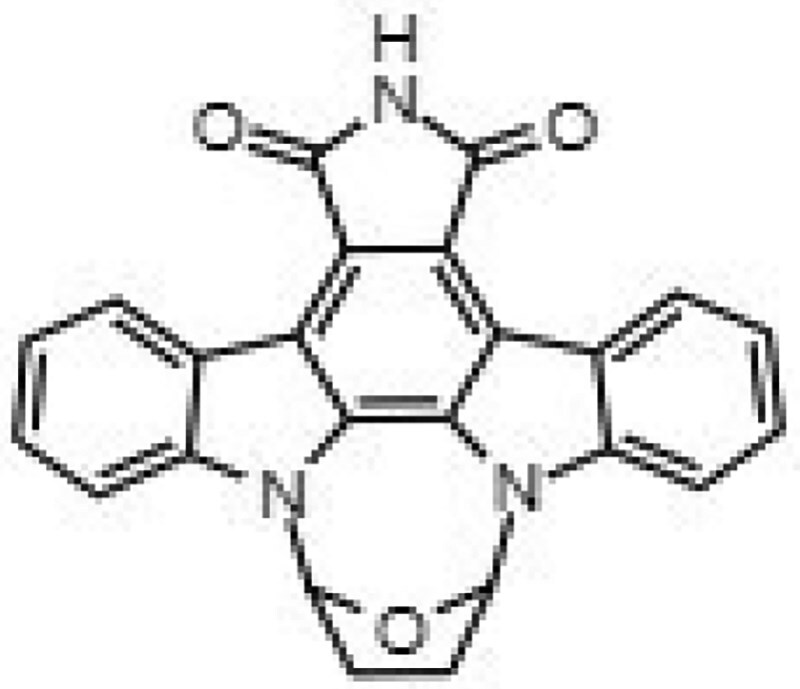	72.2 [32.9, ∼100]	0.160 [<0.01, 0.373]	no effect	NA	>32	>32
K-252a	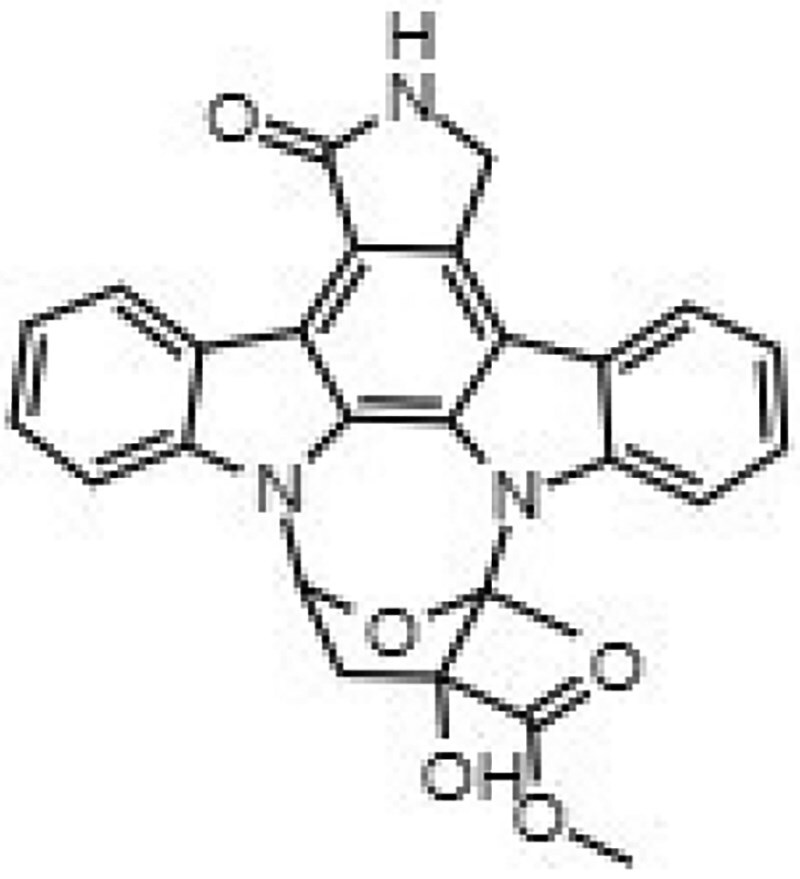	50.9 [22.5, 79.3]	0.962 [<0.01, >32]	67.5 [63.2, 71.7]	0.645 [0.526, 0.705]	1.59 [1.35, 1.86]	>32
Gö 6976	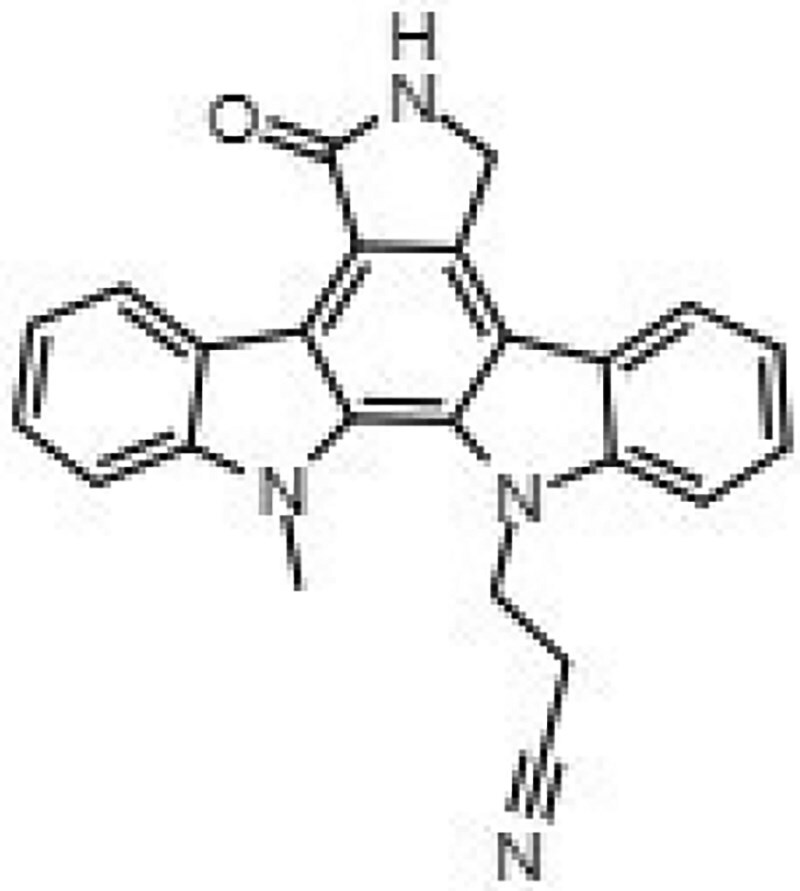	28.8 [14.2, 43.3]	1.09 [<0.01, >32]	63.4 [18.9, ∼100]	0.0891 [<0.01, 0.237]	>32	>32
arcyriaflavin A	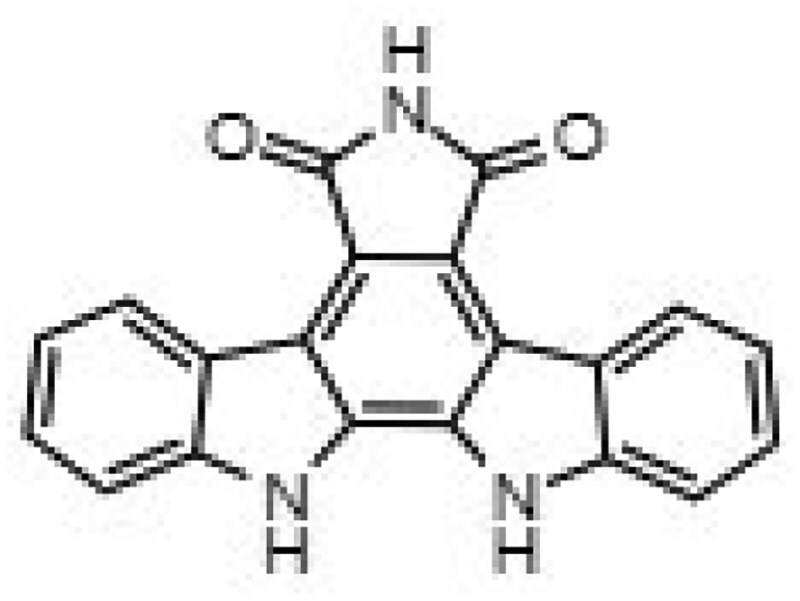	no effect	ND	31.1 [∼100, 63.4]	4.89 [<0.01, 15.2]	>32	>32
7-OH staurosporine	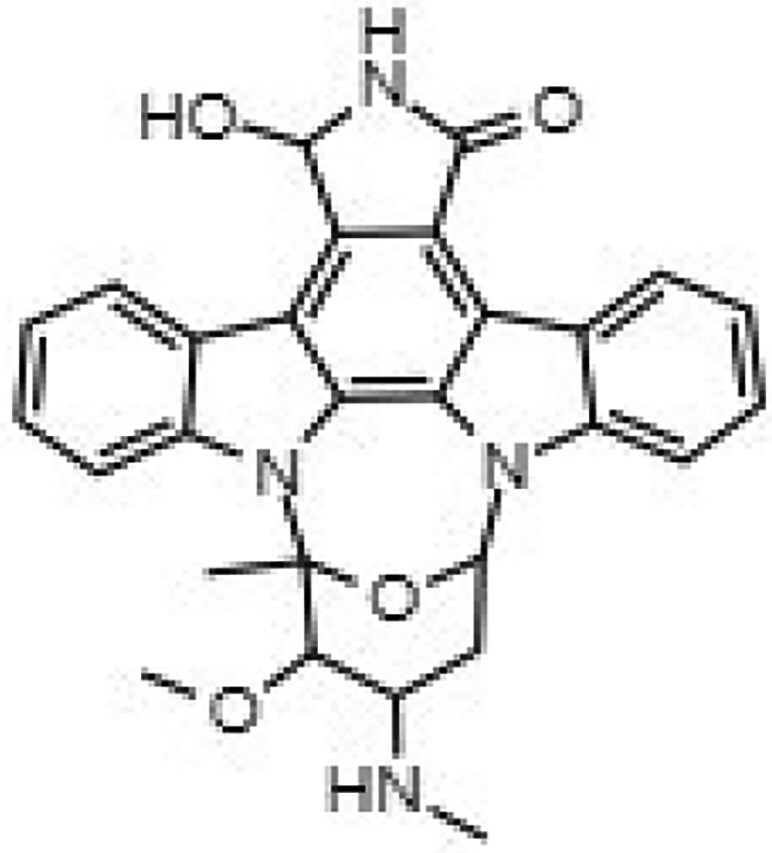	no effect	ND	28.6 [2.84, 54.4]	4.73 [<0.01, 13.6]	0.438 [0.355, 0.535]	6.25 [2.16, ND]
staurosporine	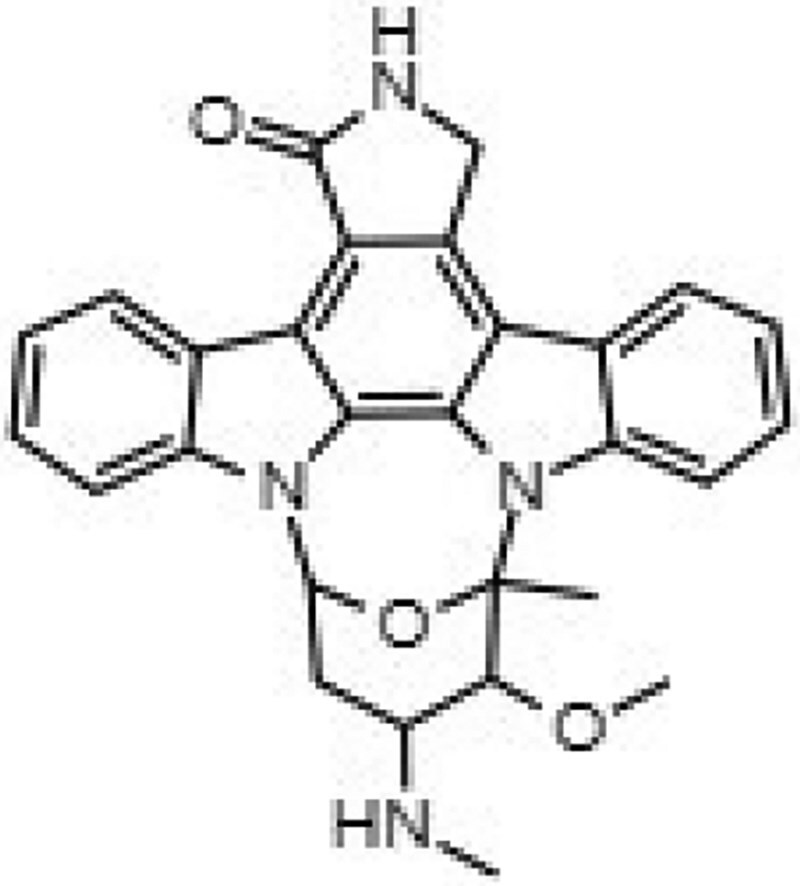	detrimental effect	ND	67.9 [6.90, ∼100]	9.26 [<0.01, 24.1]	0.246 [0.223, 0.270]	>32

Numbers between brackets represent the lower and upper limits of the 95% confidence intervals.

Several compounds also displayed antimicrobial activity against planktonic cultures, indicating that these compounds might act more like conventional antibiotics rather than HDT. However, GW296115X and SB-218078 were distinctly more effective against intracellular bacteria, with SB-218078 showing no detectable activity against planktonic bacteria, strongly indicating that the compounds act in a host-directed manner (Figure 2).

### SB-218078 and GW296115X are well-tolerated by host cells, although SB-218078 reduced host metabolic activity

**Figure 3.**
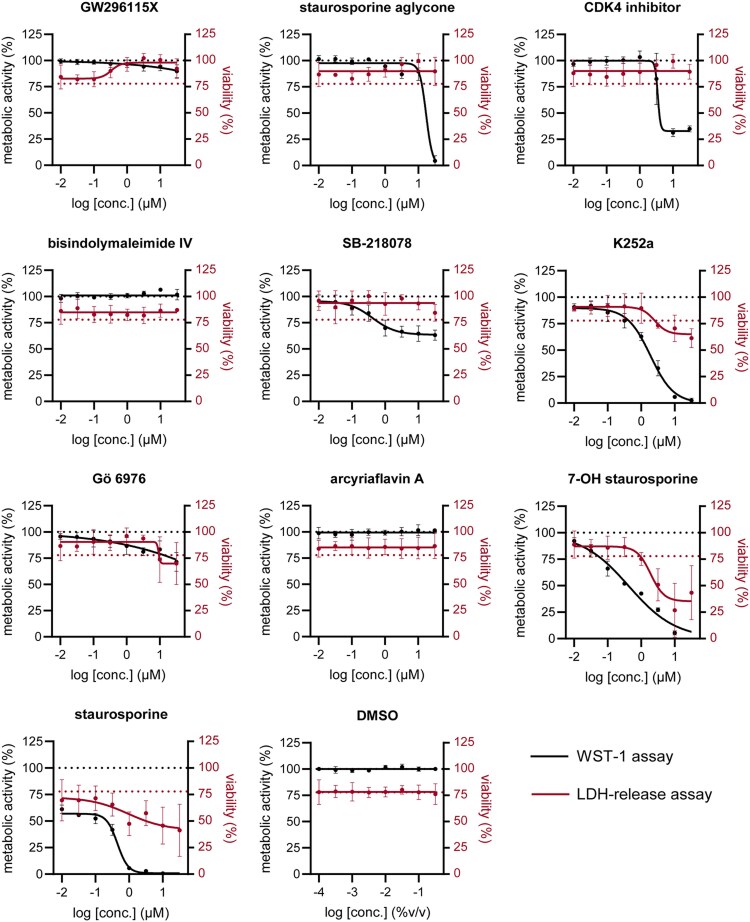
Cytotoxicity of indolocarbazoles. The effect of the compounds on host cell metabolic activity was assessed in uninfected HeLa cells using the WST-1 assay (black; *n* = 4). The metabolic activity of HeLa cells was normalized to DMSO controls. The effect of the compounds on host cell viability was assessed from the supernatant of MRSA-infected HeLa cells using the LDH-release assay (red; *n* = 3). The dotted red line depicts loss in host cell viability due to MRSA infection (i.e. baseline). Datapoints and error bars represent the mean ± SD. The curves show the result of four-parameter logistic regression analyses.

All compounds were evaluated for their effects on HeLa cell viability and metabolic activity, using LDH-release and WST-1 assays, respectively (Figure 3; Table 1). Uninfected cells were used for the WST-1 assay, as MRSA metabolic activity strongly influenced the assay’s outcome (not shown). Some compounds, including K252a, 7-OH staurosporine and staurosporine, caused notable drops in host cell viability, with LDH-release increases of 38.8%, 64.8% and 58.6%, respectively. Notably, these compounds are known inducers of apoptosis, a form of cell death where the plasma membrane remains intact, for which the LDH-release assay is relatively insensitive. In the WST-1 assay, K252a, 7-OH staurosporine and staurosporine led to a complete loss of host cell metabolic activity at the highest concentrations tested (32 µM), indicating a total loss of viable cells. None of the other compounds negatively impacted viability based on LDH release, and some even reduced LDH release, suggesting protection against MRSA-induced cell death as observed in untreated infected host cells. This effect was most pronounced for GW296115X, which increased host cell viability in a dose-dependent manner. Despite exhibiting limited impact on LDH release, most compounds affected metabolic activity. Only bisindolylmaleimide IV and arcyriaflavin A left metabolic activity unchanged, while GW296115X caused only minor reductions. In contrast, staurosporine aglycone (32 µM) and the CDK4 inhibitor (10 µM) caused significant drops in metabolic activity by 100% and 67%, respectively. SB-218078 caused a dose-dependent gradual loss in host cell metabolic activity up to 32%. In summary, while GW296115X analogues affected host cell metabolic activity to varying extents, overall host cell viability was largely maintained, and in some cases, such as with GW296115X itself, even improved.

### GW296115X and SB-218078 synergistically interact with antibiotics vancomycin and daptomycin in a mixed intracellular and extracellular MRSA infection model

**Figure 4.**
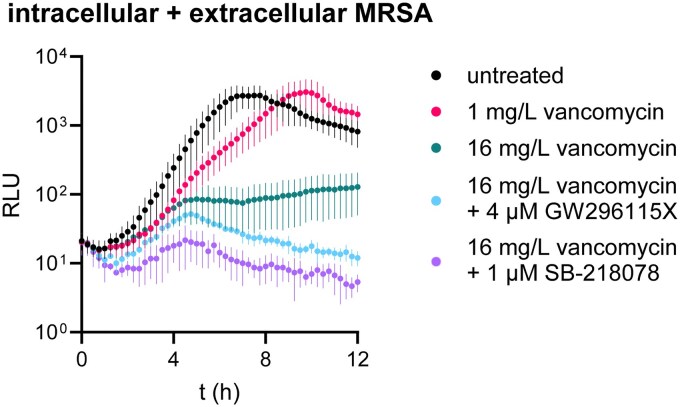
A mixed intracellular and extracellular infection model with bioluminescent MRSA that captures the antimicrobial activities of both conventional antibiotics and HDTs. HeLa cells were infected with bioluminescent MRSA after which extracellular bacteria were removed and treatments were added. The absence of gentamicin enables the bacteria to grow extracellularly upon lysis of infected host cells. The datapoints and error bars show the mean ± SD of real-time bioluminescent measurements of infected cells left untreated (*n* = 7), cells treated with vancomycin alone (*n* = 7), cells treated with both vancomycin and GW296115X (*n* = 4) and cultures treated with both vancomycin and SB-218078 (*n* = 3).

To investigate how indolocarbazoles interact with conventional antibiotics in reducing the bacterial burden during MRSA infection, we modified the infection model by omitting low-dose gentamicin during incubation. After infecting cells with MRSA, the cells were briefly treated with 30 mg/L gentamicin and thoroughly washed to remove residual gentamicin, which prevented extracellular MRSA outgrowth in previous experiments (Figure 1a). Additional washes enabled bacterial escape from HeLa cells, increasing RLUs due to extracellular growth (Figure 4). Again, we observed that intracellular bacterial growth saturated approximately 5 h after infection, which is evident from the clear difference between untreated cultures and cultures treated with 16 mg/L vancomycin. Interestingly, while 1 mg/L vancomycin initially prevented bacterial outgrowth, it failed after 5 h. Although 16 mg/L vancomycin alone reduced MRSA growth, addition of GW296115X or SB-218078 was required to achieve a declining bacterial load.

**Figure 5.**
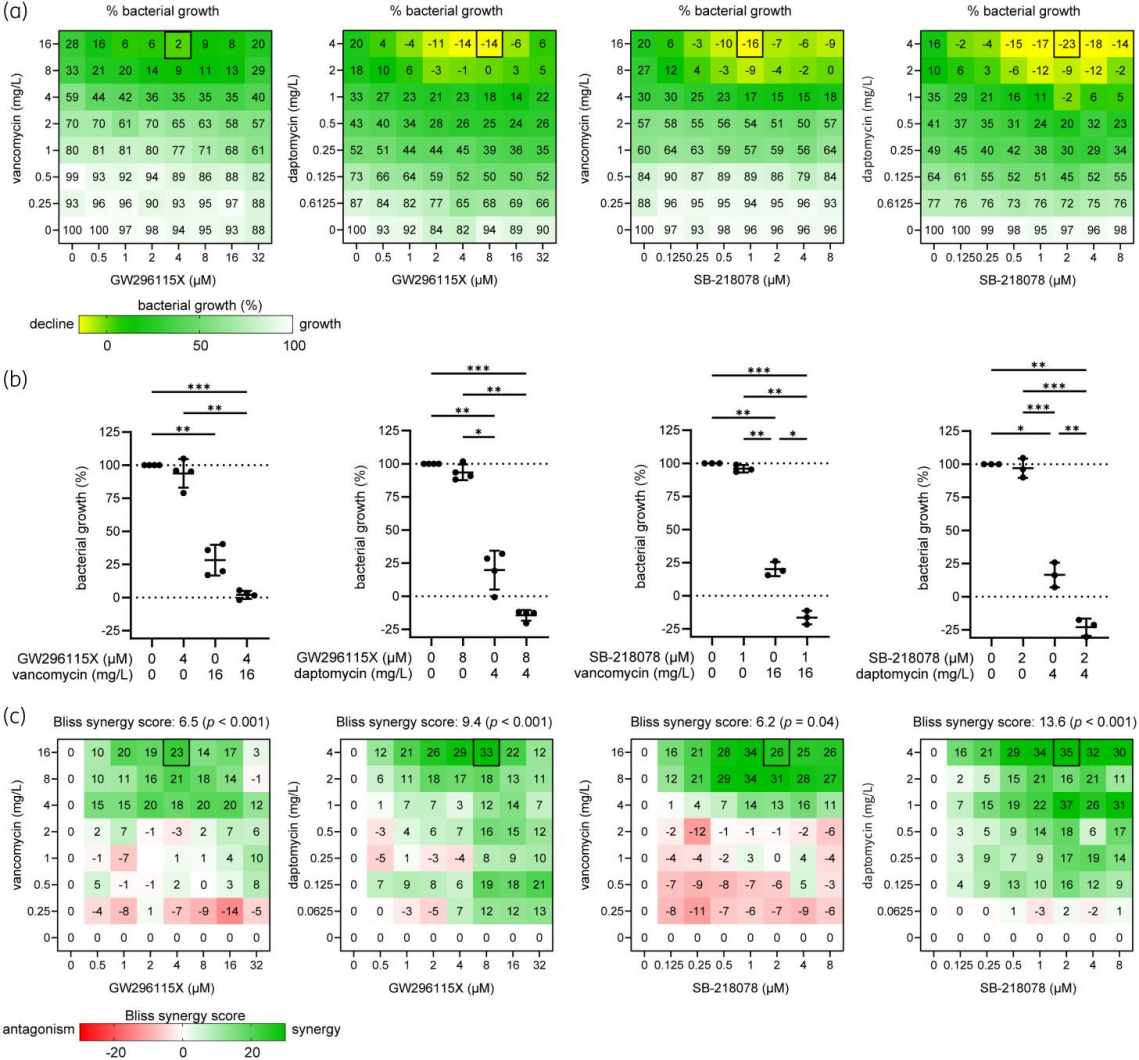
Interaction between indolocarbazole HDTs and conventional antibiotics. (a) MRSA-lux-infected HeLa cells in a mixed intracellular and extracellular infection model were treated with chequerboard titrations of GW296115X versus vancomycin (*n* = 4), GW296115x versus daptomycin (*n* = 4), SB-218078 versus vancomycin (*n* = 3) and SB-218078 versus daptomycin (*n* = 3). Numbers depict the average percentual bacterial growth until a maximum bioluminescent signal was reached for the untreated control. A negative number depicts a decline of bioluminescent signal, corresponding to bacterial inhibition. (b) Bart chart showing the antimicrobial effect of single drugs and drug combinations at their most effective concentrations in combination, based on the data from (a). Error bars show the mean + SD. The data were tested for significant differences between groups using a one-way ANOVA test for matched samples and Tukey’s multiple comparisons *post hoc* test (*P < 0.05; **P < 0.01; ***P < 0.001). (c) The data from (a) were used to determine the interaction between indolocarbazole HDTs and conventional antibiotics by calculating the Bliss synergy score for each combination of drugs using the SynergyFinder+ web application. Synergy scores for each combination of drug concentrations as well as an overall synergy scores are depicted. The *P* values of the overall bliss synergy scores show the statistical significance of the difference between the estimated average synergy score and the expected synergy score of zero (i.e. no interaction) with a *P* value of <0.05 being statistically significant.

To examine HDT-antibiotic interactions, we performed chequerboard titrations of GW296115X or SB-218078 with vancomycin or daptomycin. Bacterial growth rates were averaged and normalized to untreated controls. Consistent with our previous findings (Figure 1b), neither 16 mg/L vancomycin nor 4 mg/L daptomycin alone was able to fully eliminate the bacteria or completely inhibit their growth (Figure 5a). Co-treatment with 4 µM GW296115X and 16 mg/L vancomycin fully suppressed bacterial growth, whereas vancomycin alone allowed 28.3% residual growth. Notably, the addition of ≥1 µM GW296115X to 4 mg/L daptomycin resulted in bacterial killing. Similarly, the addition of ≥125 nM SB-218078 to 16 mg/L vancomycin, or ≥250 nM SB-218078 to 4 mg/L daptomycin, also led to declining bacterial loads, and these combinations significantly outperformed antibiotic treatment alone (Figure 5b).

To corroborate our bioluminescence data, endpoint cfu counts were included for antibiotic treatment alone and in combination with HDTs (Figure S3). These data confirmed that reductions in bioluminescence were accompanied by corresponding reductions in viable bacterial load. While differences were not statistically significant due to inter-experimental variation, cfu counts were consistently lower with adjunctive treatment using GW296115X or SB-218078 compared to vancomycin or daptomycin alone. This pattern was consistently observed across both HeLa (Figure S3a) and MCF-7 (Figure S3b) host cell models infected with MRSA LAC JE2 strain, as well as in HeLa cells infected with other *S. aureus* strains, including LUH15392 (Figure S3c), LUH15393 (Figure S3d) and Newman (Figure S3e).

Based on the bioluminescent data, antibiotics and indolocarbazoles appeared to synergistically target extracellular and intracellular bacteria, respectively. Bliss synergy scores confirmed statistically significant interactions (Figure 5c). The strongest synergy was observed for daptomycin/SB-218078 (overall score: 13.6; 4 mg/L daptomycin + 2 µM SB-218078: 35.1). Interestingly, vancomycin demonstrated synergistic effects with indolocarbazoles only at concentrations ≥ 4 mg/L, while daptomycin exhibited synergism at lower concentrations (e.g. 125 μg/L). Overall, these findings highlight the potential of combining indolocarbazoles with conventional antibiotics to enhance antibacterial efficacy through synergistic mechanisms, offering a promising strategy for overcoming bacterial resistance.

## Discussion

Invasive strains of *S. aureus*, especially MRSA, are challenging to treat due to their persister phenotype with high antibiotic tolerance and their ability to evade antibiotics by growing intracellularly.^3–8^ In this study, we explored GW296115X analogues as adjunctive HDT, building on the previous identification of GW296115X as an effective HDT.^11^ Consistent with previous research, conventional antibiotics like gentamicin and vancomycin showed limited efficacy against intracellular MRSA, while daptomycin and tetracycline retained partial activity.^9,20,21^ Interestingly, linezolid’s intracellular activity was largely maintained, aligning with some previous studies^8^ but differing from others^9,22^—potentially due to variations in host cell types and bacterial strains. Overall, these findings underscore the need for alternative strategies to combat intracellular infections.

Several indolocarbazoles, notably GW296115X and SB-218078, demonstrated potent activity against intracellular MRSA with minimal impact on host cell viability. These compounds exhibited little activity against planktonic MRSA, reinforcing their role as HDTs. Importantly, GW296115X and SB-218078 demonstrated synergy with antibiotics like vancomycin and daptomycin at clinically relevant concentrations, significantly enhancing bacterial inhibition. Therefore, combining indolocarbazole HDTs with conventional antibiotics may offer an effective approach to treat infection with invasive strains of (antibiotic-resistant) *S. aureus*.

While endpoint cfu measurements provided additional support for the efficacy of combined treatment (Figure S3), the observed differences between antibiotic monotherapy and HDT-antibiotic combinations did not reach statistical significance. This likely reflects the timing of sampling, as bacterial growth had entered the stationary or decline phase according to the bioluminescent signal—potentially due to host cell lysis and the resulting loss of the intracellular niche. In contrast, bioluminescence provided a dynamic, non-destructive readout that enabled the detection of short-term treatment effects of the HDTs during early intracellular growth of MRSA, while showing a strong correlation with bacterial load at these initial stages of infection (Figure S1). This approach allowed treatment effects to be evaluated *in situ* over time, which is particularly valuable for capturing the early action of HDTs prior to the later effects of antibiotics.

SB-218078 emerged as approximately four times more potent than GW296115X in targeting intracellular MRSA. Initially identified as a protein kinase C (PKC) inhibitor with potency comparable to staurosporine, SB-218078 was later described to be selective for human checkpoint kinase 1 (Chk1), which may be attributed to differences in the PKC isoforms tested.^23,24^ Comprehensive kinase selectivity profiling has since demonstrated that SB-218078 exhibits slightly better selectivity than staurosporine, a known promiscuous kinase inhibitor.^25^ The same is true for staurosporine aglycone, which was also found active against intracellular MRSA. Nevertheless, SB-218078 and staurosporine aglycone were much better tolerated in our cellular assays than for instance staurosporine or 7-hydroxy staurosporine. Bisindolylmaleimide IV and CDK4 inhibitor, which have a more selective kinase inhibition profile, were also found effective but exhibited lower potency than GW296115X.^25^

Prior studies suggest that indolocarbazole potency and selectivity stem from interactions within the kinase catalytic pockets. The indolocarbazole or bisindole cores mimic adenine within the ATP-binding pocket, while lactam, 7-hydroxylactam or maleimide moieties are important determinants of specificity by interacting with the kinase hinge region.^26,27^ Interestingly, intracellular MRSA was inhibited by indolocarbazoles possessing lactam (e.g. staurosporine or K-252a) or maleimide moieties (e.g. GW296115X), but not by 7-hydroxystaurosporine possessing a 7-hydroxylactam moiety. The lack of activity of arcyriaflavin A, despite having the maleimide moiety, is likely the result of its poor solubility and/or poor cell penetrance, as previously suggested.^14^ In line with previous studies, a partial loss in potency was observed for bisindolylmaleimide, which, unlike indolocarbazoles, possesses free-rotating ring structures that results in lower affinity interactions.^14,28^ Additionally, sugar moieties in staurosporine, 7-hydroxystaurosporine, K-252a and SB-218078 mimic ribose within the ATP-binding pocket, enhancing target affinity but reducing selectivity. Staurosporine, 7-hydroxystaurosporine and K-252a demonstrated high cytotoxicity, as evidenced by LDH-release assay and WST-1 assay. In contrast, SB-218078 demonstrated significantly lower cytotoxicity, with only a moderate reduction in HeLa cell metabolic activity, likely owing to the maleimide moiety’s positive influence on kinase selectivity.^25,29^ The superior selectivity of GW296115X is likely due to the presence of the methoxy groups that are attached to the indolocarbazole ring system. Therefore, integrating the beneficial properties of SB-218078 and GW296115X will be an important next step that may lead to the development of an indolocarbazole with enhanced potency relative to GW296115X, while maintaining its selectivity. Furthermore, future studies should address the relatively narrow concentration window within which GW296115X—and to a lesser extent, SB-218078—exert maximal activity against intracellular MRSA (Figure 5a). At higher concentrations, a partial decline in efficacy is observed, potentially due to off-target kinase inhibition that may interfere with their host-directed therapeutic effects.

Although HeLa cells are frequently used as host cell type for *S. aureus* intracellular infection models due to their practicality and robustness, they are a simplification of the diversity of cell types that *S. aureus* is able to infect. In previous work, we assessed GW296115X activity across a panel of cell lines representing diverse tissue origins, including MCF-7, MelJuSo, A549 and PMA-differentiated THP-1 cells.^11^ GW296115X was effective in HeLa, MCF-7 and MelJuSo cells, and also reduced MRSA-induced cytotoxicity in A549 cells, indicating broader host-directed effects. In the present study, we extend these findings by showing that SB-218078 exhibits similar activity to GW296115X in both HeLa and MCF-7 cells (Table 1, Figure 2, Figure S3). These observations suggest that the activity of these compounds is not restricted to a single cell type. To support clinical translation, future studies should incorporate more physiologically relevant systems—such as primary human cells or *in vivo* models—that better reflect the diverse tissue tropism and pathological manifestations of *S. aureus* infections.

In summary, SB-218078 and GW296115X exhibit potent host-directed activity against intracellular MRSA, while showing minimal effects on host cell viability. Furthermore, both compounds displayed synergistic interactions with clinically relevant antibiotics vancomycin and daptomycin. Integrating the strengths of indolocarbazoles, along with optimizing their pharmacokinetics, could lead to an effective adjunctive therapy against intracellular MRSA and help to overcome antibiotic resistance.

## Supplementary Material

dkaf198_Supplementary_Data

## Supplementary data

Figures S1–S3 are available as Supplementary data at *JAC* Online.
