# Supplementary material for: Indolocarbazoles as host-directed therapeutics against intracellular infections by methicillin-resistant Staphylococcus aureus
Source: J Antimicrob Chemother. 2025 Jul 1;80(8):2257–68. doi: 10.1093/jac/dkaf198 (PMC12313463; doi:10.1093/jac/dkaf198)
Supplement: dkaf198_Supplementary_Data [file dkaf198_supplementary_data.docx]

**
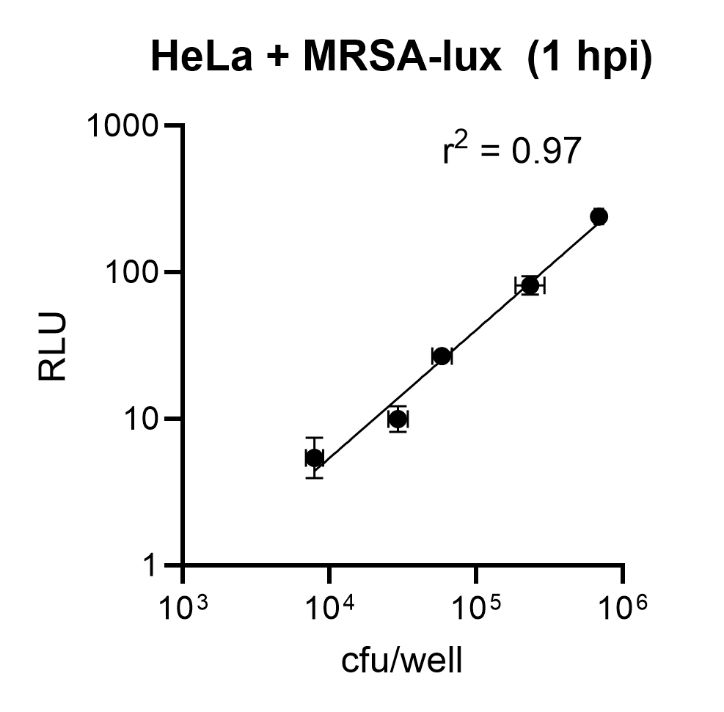
**

**Figure S1** Pearson’s correlation between RLU and cfu for intracellular MRSA infection. HeLa cells were infected with MRSA-lux at MOI 3, 10, 30, 100 or 300 for 1 h. Thereafter, extracellular bacteria were killed by 15 min incubation in the presence of 30 µg/mL gentamicin, followed by 1 h incubation in the presence of 5 µg/mL gentamicin. Bioluminescence was measured and the host cells were lysed immediately thereafter. The cfu counts in the lysates were determined and correlated with RLUs.

**

**

**Figure S2** Overlap between bioluminescence and turbidity of planktonic cultures of MRSA-lux as measurements of growth after 18 hours of treatment with antibiotics tetracycline (left) and linezolid (right). The turbidity was measured at 600 nm and background subtraction was performed (ΔOD600). The curves were plotted using 4-parameter logistic regression analyses.

**
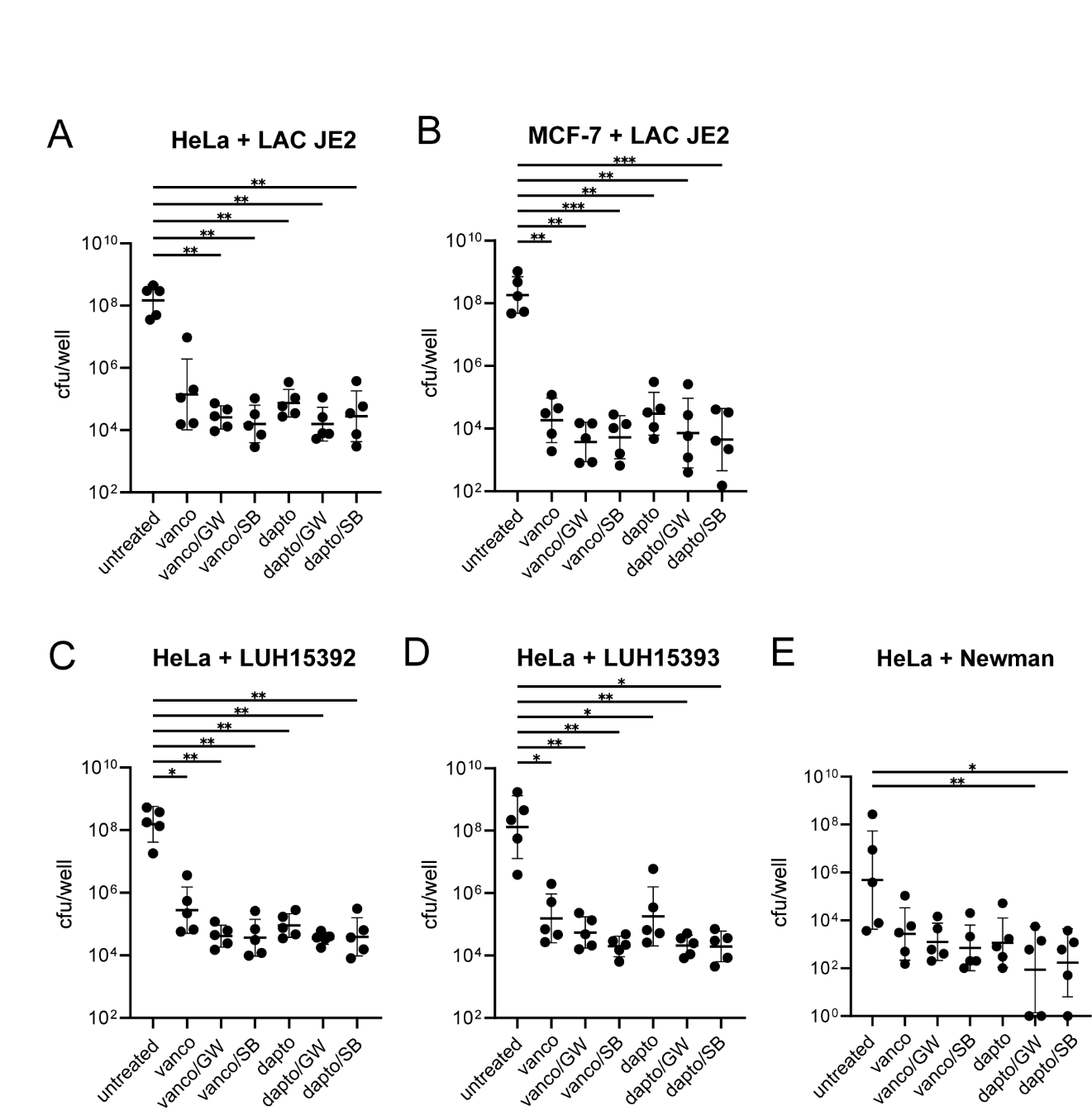
**

**Figure S3** The antimicrobial activity of HDTs, antibiotics and combinations upon infection of HeLa and MCF-7 cells with different strains of *S. aureus*. **A.** HeLa cells were infected with MRSA USA300 LAC JE2 (MOI = 10) and treated for 24 h with 16 µg/mL vancomycin (vanco), 4 µg/mL daptomycin (dapto), 4 µM GW296115X (GW), 1 µM SB-218078 (SB), or combinations thereof. **B.** Same as A., but with MCF-7 cell line as host cells. **C-E.** Same as A., but with LUH15392, LUH15393 and Newman *S. aureus* strains. Error bars show the mean + SD of log_10_-transformed data. The log_10_-transformed data was tested for significant differences between groups using a one-way ANOVA test for matched samples and Tukey’s multiple comparisons *post hoc* test (**p* < 0.05; ** *p* < 0.01; ****p* < 0.001).
